# Tim-3 protects decidual stromal cells from toll-like receptor-mediated apoptosis and inflammatory reactions and promotes Th2 bias at the maternal-fetal interface

**DOI:** 10.1038/srep09013

**Published:** 2015-03-11

**Authors:** SongCun Wang, ChunMei Cao, HaiLan Piao, YanHong Li, Yu Tao, XiaoMing Zhang, Di Zhang, Chan Sun, Rui Zhu, Yan Wang, MinMin Yuan, DaJin Li, MeiRong Du

**Affiliations:** 1 Laboratory for Reproductive Immunology, Hospital and Institute of Obstetrics and Gynecology, Fudan University Shanghai Medical College, Shanghai 200011, China; Shanghai Key Laboratory of Female Reproductive Endocrine Related Diseases, Shanghai, 200011, China; 2 Cancer Institute, Fudan University Shanghai Cancer Center, Shanghai, 200032, China; 3 Unit of Innate Defense and Immune Modulation, Key Laboratory of Molecular Virology and Immunology, Institute Pasteur of Shanghai, Chinese Academy of Sciences, Shanghai 200025, China

## Abstract

Toll-like receptors (TLRs) are important in mediating immune responses against various pathogens during pregnancy. However, uncontrolled TLR-triggered inflammation will endanger normal pregnancy, resulting in pregnancy loss. Therefore, maintenance of a moderate inflammatory response is crucial for successful pregnancy under conditions of infection. Here, we demonstrated significantly lowered expression of T-cell immunoglobulin and mucin domain 3 (Tim-3) in miscarried decidual stromal cells (DSCs), indicating that Tim-3 might play important roles in maintaining successful pregnancies. Activation of TLR signaling induced pro-inflammatory cytokine production and apoptosis of DSCs, which was accompanied by up-regulated Tim-3 expression. Tim-3, in turn, protected DSCs from TLR-mediated apoptosis in an ERK1/2 pathway-dependent manner. In addition, Tim-3 inhibited TLR signaling-induced inflammatory cytokine production by DSCs through suppressing NF-κB activation. Tim-3 increased production of T helper 2 (Th2)-type cytokines by DSCs and reversed the inhibitory effect of LPS on Th2 cytokine generation by up-regulation of interferon regulatory factor 4 expression. Tim-3 blockade abolished the effect of Tim-3 on the inflammatory response to LPS stimulation. Thus, Tim-3 signaling could represent a “self-control” mechanism in TLR-triggered inflammation during pregnancy. These findings identify Tim-3 as a key regulator of DSCs and suggest its potential as a target for the treatment of spontaneous abortion.

Miscarriage is the most common complication of pregnancy. However, in most cases the etiology is unknown^1^. Sophisticated mechanisms are required for the maintenance of pregnancy. For years, the pattern of immune regulation during pregnancy has been based on the T helper 2 (Th2) bias at the maternal-fetal interface^2^. Increased inflammatory response in the decidua is frequently reported in women suffering recurrent pregnancy losses. Moreover, maternal administration of the Th2 cytokine, or blockade of the Th1 cytokine prevents pregnancy loss induced by lipopolysaccharide (LPS)^3^,^4^. During early pregnancy, the developing decidua undergoes dramatic changes in response to invading trophoblasts. Decidual stromal cells (DSCs) are the predominant cell type of the maternal decidua and play a key role in embryo implantation and placentation^5^. Apart from nutritive and endocrine functions, DSCs are believed to be involved in many immune activities, such as cytokine production and antigen presentation, and regulate the decidual immune responses that may lead to either a successful pregnancy or miscarriage^6^,^7^.

In the embryo allograft rejection, infection represents an important triggering event, and uncontrolled inflammation is harmful to pregnancy which may lead to miscarriage. Toll-like receptors (TLRs) are a family of innate pattern recognition receptors that recognize pathogen-associated molecules and activate antipathogen responses, such as expression of pro-inflammatory cytokines^8^. Among the TLR family, TLR2 has the widest microbial specificity and TLR3 plays important roles in responses to viruses, whereas TLR4 recognizes gram-negative LPS^9^. Overshooting of TLR signaling is associated with miscarriage. Binding of TLRs often results in production of inflammatory cytokines through activation of the NF-κB pathway^10^, which is detrimental to pregnancy. Administration of poly (I:C), a ligand of TLR3, induces abortion in various murine mating pairs^10^. LPS-mediated pregnancy loss has been widely studied. It has been reported that LPS administration can increase levels of pro-inflammatory molecules and nitric oxide synthesis, inducing deficient uteroplacental perfusion and macrophage dysregulation^3^,^10^,^11^,^12^. Neutralization of LPS or blockage of TLR4 signaling prevents fetal loss in murine pregnancy^13^.

T-cell immunoglobulin and mucin domain 3 (Tim-3) is a newly identified regulatory molecule. Upon interaction with its ligand (e.g., galectin-9), Tim-3 mediates phagocytosis of apoptotic cells and terminates T helper 1 (Th1) and T cytotoxic 1 cell (Tc1) responses by inducing immune cell apoptosis, which play critical roles in regulating immune activities during infection, tumor growth, and organ transplantation^14^. Though it has been regarded as an inhibitory molecule, Tim-3 may synergize with the TLR system to influence inflammatory conditions by promoting or terminating Th1 immune activity^15^. Thus, Tim-3 may serve various, possibly opposing roles. Tim-3 can suppress TLR4 signal-mediated inflammatory reactions and damage by inhibiting NF-κB activation^16^,^17^. Expression of Tim-3 and TLR4 is reciprocally regulated^18^,^19^. To date, however, a detailed description of the communication between Tim-3 and TLRs at the maternal-fetal interface remains incomplete.

In the present study, we found that Tim-3 was expressed at a higher level in DSCs from normal pregnancy than those from miscarriage, suggesting a new potential diagnostic biomarker for successful pregnancy. Although there was no difference in TLR expression between normal pregnancy and miscarriage, the enhanced Tim-3 expression might reduce TLR-trigged pro-inflammatory cytokine production and apoptosis of DSCs through activation of ERK1/2 pathway and subsequent inhibition of NF-κB. In addition, Tim-3 promoted Th2 bias at the maternal-fetal interface accompanied by up-regulated immune regulatory factor (IRF) 4 expression, whereas blockade of Tim-3 signaling by anti–Tim-3 monoclonal antibody (mAb) abolished the protective effects of Tim-3. Thus, Tim-3 represents a negative feedback mechanism employed by DSCs to avoid overshooting the inflammatory reaction, which might be helpful in preventing excess inflammatory response-induced pregnancy loss.

## Results

### Tim-3 down-regulation correlated with human miscarriage

**Figure 1:**
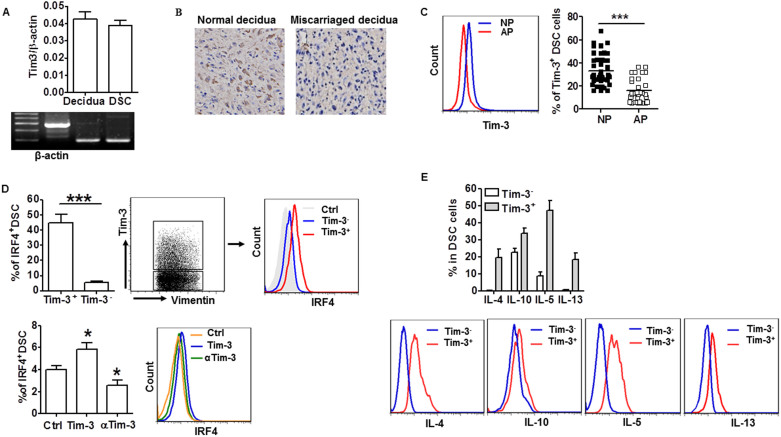
Tim-3 downregulation correlated with human miscarriage. (A)Real-time PCR analysis of Tim-3 mRNA in human first trimester decidual tissue and DSCs. β-actin served as internal control. (B) Immunohistochemical localization of Tim-3 in decidual tissue from normal pregnancy and miscarriage. (C) Flow cytometric analysis (left) and quantitation (right) of Tim-3 expression in DSCs from normal pregnancy (n = 48) and miscarriage (abnormal pregnancy; n = 32). NP: normal pregnancy; AP: abnormal pregnancy. (D) Flow cytometric analysis and quantitation of IRF4 expression in Tim-3^+^ and Tim-3^−^ DSCs (upper) and in DSCs in normal pregnancy treated with Tim-3, anti-Tim-3 mAb, or isotype-control IgG (Ctrl); n = 12 (lower). (E) Flow cytometric analysis (lower) and quantitation (upper) of production of the indicated Th2-type cytokines by Tim-3^+^ and Tim-3^−^ DSCs of normal pregnancy; n = 18. Data represent mean ± standard error of the mean (SEM). The representative flow cytometry plots are from one representative experiment. *P ≤ 0.05, ***P ≤ 0.001.

Tim-3 is an important immune regulatory molecule. To investigate whether the Tim-3 pathway is also involved in maternal-fetal tolerance, we first examined the expression of Tim-3 in deciduae from human first trimester pregnancy, and whether there was a correlation between Tim-3 expression and pregnancy outcome. As shown in Figure 1A, 1B and 1C, both Tim-3 mRNA and protein were detected in both decidual tissue and DSCs. Interestingly, a higher level of Tim-3 expression was observed in decidual tissue (Figure 1B) and DSCs (Figure 1C) from normal pregnancy than from miscarriage. Because Th2 bias at the maternal-fetal interface is crucial for maintenance of normal pregnancy, and because interferon regulatory factor 4 (IRF4) is a transcription factor important for Th2 bias, we further investigated the correlation between Tim-3 signal and IRF-4 expression^20^. Figure 1D (upper) shows notably increased IRF4 expression in Tim-3^+^ DSCs. Furthermore, treatment of DSCs with Tim-3 markedly up-regulated the expression of IRF4, whereas anti–Tim-3 mAb treatment down-regulated IRF4 expression in DSCs (Figure 1D, lower). We then directly measured Th2 cytokine production by Tim-3^+^ and Tim-3^−^ DSCs. Consistently, Tim-3^+^ DSCs produced higher levels of Th2 cytokines than Tim-3^−^ DSCs (Figure 1E). These findings suggest a correlation between low levels of Tim-3 and spontaneous abortion.

### TLR pathways induced apoptosis and pro-inflammatory cytokine production by DSCs

**Figure 2:**
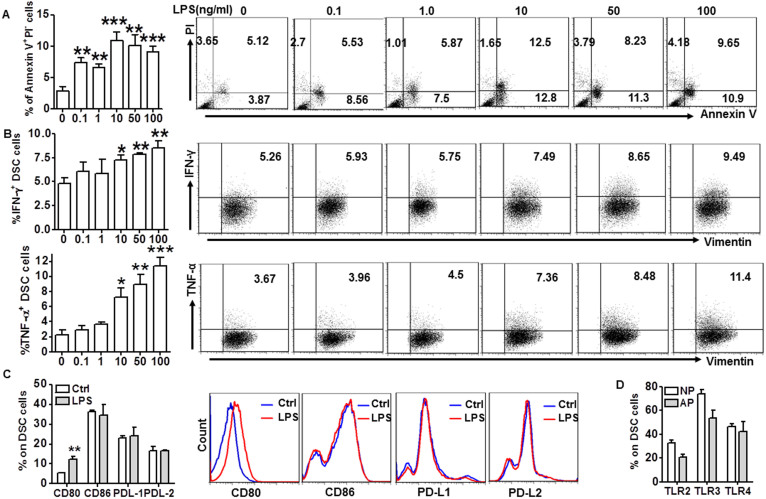
TLR pathways induced apoptosis and proinflammatory cytokine production in DSCs. (A and B) Flow cytometric analysis (right) and quantitation (left) of apoptosis based on annexin expression and PI staining (A) and IFN-γ and TNF-α production (B) in DSCs treated with the indicated concentrations of LPS. (C) Flow cytometric analysis (right) and quantitation (left) of production of the indicated cytokines by DSCs treated with or without (Ctrl) 10 ng/ml LPS. (D) Quantitation of flow cytometric analysis of expression of the indicated TLRs in DSCs from normal pregnancy (n = 51) and miscarriage (n = 21). Data represent mean ± SEM. Flow cytometry plot is from one representative experiment. *P ≤ 0.05, **P ≤ 0.01, ***P ≤ 0.001.

Excessive activation of TLRs is associated with miscarriage^10^,^11^,^13^, but most of those studies focused on immune cells. Because DSCs also play an important role in pregnancy maintenance, we investigated the effect of TLR signaling activation on DSCs and pregnancy outcome. We found that ligands of different TLRs, including pGN-SA (ligand of TLR2), poly(I:C) (ligand of TLR3), and LPS (ligand of TLR4), induced apoptosis of DSCs (Figure 2A and Figure S1A), and increased DSC production of pro-inflammatory Th1 cytokines IFN-γ and TNF-α (Figure 2B and Figure S1B). The apoptosis was further confirmed by detecting caspase 3 activation and nuclear condensation (Figure S1C). All these effects were observed when DSCs were treated with different ligands at a concentration of 10 ng/ml; therefore, we used this concentration in the follow-up experiments. We also measured the secretion of IFN-γ and TNF-α by DSCs treated with LPS (10 ng/ml) for different times. The results in Figure S1D showed that LPS stimulation increased pro-inflammatory cytokine secretion in a time-dependent manner. In addition to inducing apoptosis and Th1 cytokine production in DSCs, LPS also promoted CD80 expression in DSCs, but had no effect on CD86 and the inhibitory co-stimulatory molecules PDL-1 and PDL-2 levels (Figure 2C). Thus, activation of TLR signals might disrupt DSC homeostasis, forming an inflammatory microenvironment at the maternal-fetal interface. Unexpectedly, there was no significant difference in expression levels of TLRs in DSCs from normal pregnancy and miscarriage (Figure 2D). This raised the question of what mechanism protects DSCs from TLR-mediated apoptosis and pro-inflammatory reaction.

### Tim-3 protected DSCs from TLR-mediated apoptosis through activation of the ERK1/2 pathway

**Figure 3:**
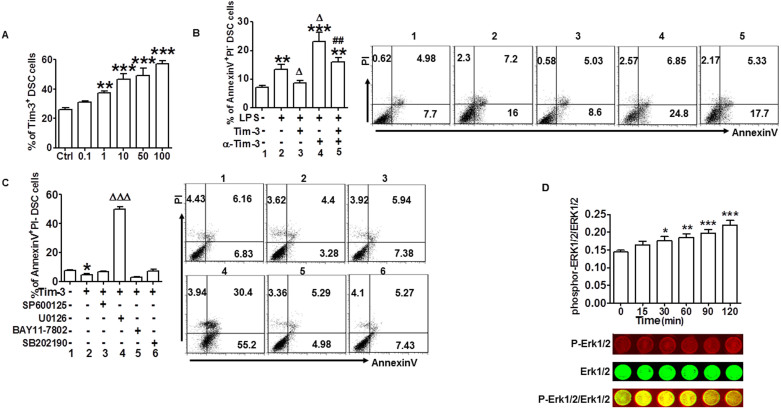
Tim-3 protected DSCs from TLR-mediated apoptosis through activation of the ERK1/2 pathway. (A) Quantitation of flow cytometric analysis of Tim-3 expression in DSCs stimulated with the indicated concentrations of LPS. (B) Flow cytometric analysis (right) and quantitation (left) of apoptosis based on annexin expression and PI staining in DSCs stimulated with LPS (10 ng/ml) in the presence or absence of Tim-3 and anti–Tim-3 mAb. Treatment group numbers below histogram bars correspond to numbers on panels of flow cytometry plot. (C) Flow cytometric analysis (right) and quantitation (left) of apoptosis in DSCs after treatment with Tim-3 in the presence or absence of the indicated inhibitors of signal transduction. (D) In-cell western analysis (lower) and quantitation (upper) of the level of ERK1/2 phosphorylation relative to total ERK1/2 in DSCs treated with Tim-3 for the indicated times. Images are representative of six individual experiments. Data represent mean ± SEM. Flow cytometry plot is from one representative experiment; n = 18 DSC cells in the first trimester of normal pregnancy. *P < 0.5, **P < 0.01, ***P < 0.001, compared with group 1; ΔP < 0.5, ΔΔP < 0.01, ΔΔΔP < 0.001, compared with group 2; #P < 0.5, ##P < 0.01, ###P < 0.001, compared with group 3.

As noted previously, Tim-3 is involved in the regulation of TLR signal-mediated inflammatory reaction and damage^15^,^16^,^17^. Given that Tim-3 expression in DSCs was significantly decreased in miscarriage compared with normal pregnancy, and stimulation of DSCs with ligands of different TLRs resulted in up-regulation of Tim-3 expression (Figure 3A and Figure S2A), we hypothesized that, during normal pregnancy, Tim-3 provides protection from TLR-mediated damage.

To test this hypothesis, we first investigated whether Tim-3 had an effect on TLR signal-mediated DSC apoptosis. Treatment with LPS (Figure 3B) or poly(I:C) or pGN-SA (Figure S2B) induced DSC apoptosis. Pretreatment with Tim-3 markedly inhibited the apoptosis, whereas administration of anti–Tim-3 mAb exacerbated it. If Tim-3 and anti-Tim-3 mAb were administered at the same time, their respective effects on TLR signaling-mediated apoptosis were reciprocally neutralized.

To date, no signaling pathway has been precisely implicated in the regulation of Tim-3 function. To further investigate the signaling pathways involved in Tim-3–mediated apoptosis prevention, the effect of specific signal transduction inhibitors on DSC apoptosis was examined. The results showed that most signaling pathways inhibitors, including SP600125, SB202190, and BAY 11-7082 had no effect on Tim-3–mediated apoptosis inhibition. However, U0126 (inhibitor of ERK1/2) notably reversed this effect (Figure 3C). We found that treatment with U0126 alone did not increase cell apoptosis (unpublished data), suggesting the apoptosis caused by U0126 is not due to its cytotoxic effect but a reverse of Tim-3-mediated action. Next, we analyzed whether Tim-3 activated ERK1/2 signaling in DSCs. As shown in Figure 3D, administration of Tim-3 significantly increased ERK1/2 phosphorylation, sustaining for at least 120 min. These findings suggest that Tim-3 protection of DSCs from apoptosis might depend on the ERK1/2 signaling pathway.

### Tim-3 protected DSCs from TLR-mediated pro-inflammatory response through NF-κB inhibition

**Figure 4:**
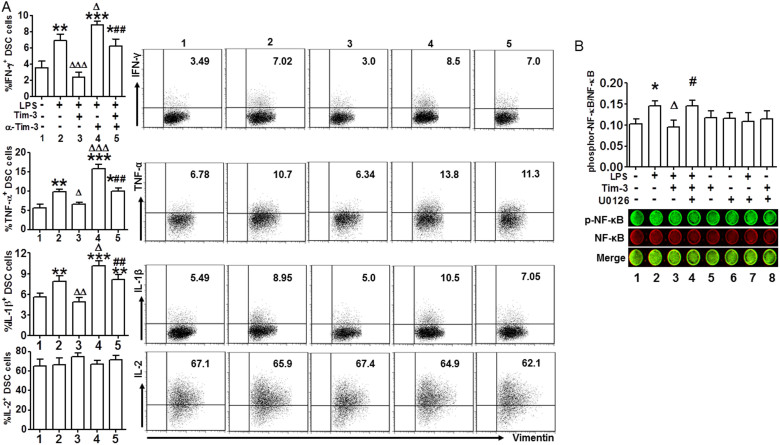
Tim 3 protected DSCs from TLR-mediated proinflammatory response via NF-κB inhibition. (A) Quantitation (left) of flow cytometric analysis (right) of IFN-γ, TNF-α, IL-1β, and IL-2 production by DSCs treated with or without LPS (10 ng/ml) in the presence or absence of Tim-3 and anti–Tim-3 mAb. Data represent mean ± SEM. Flow cytometry plot shown is from one representative experiment; n = 14 DSCs in the first trimester of normal pregnancy. Treatment group numbers below histogram bars correspond to numbers on panels of flow cytometry plot. (B) In-cell western analysis (lower) and quantitation (upper) of phosphorylation/activation of NF-κB in DSCs stimulated with or without LPS, Tim-3, and U0126, alone or together. Images are representative of three individual experiments. *P < 0.5, **P < 0.01, ***P < 0.001, compared with group 1; ΔP < 0.5, ΔΔP < 0.01, ΔΔΔP < 0.001, compared with group 2; #P < 0.5, ##P < 0.01, ###P < 0.001, compared with group 3.

To further explore the beneficial roles of Tim-3 in pregnancy, we examined pro-inflammatory cytokine production in DSCs treated with LPS, alone or together with Tim-3 and anti-Tim-3. Figure 4A shows that Tim-3 inhibited LPS-induced production of IFN-γ, TNF-α and IL-1β but not IL-2, and administration of anti–Tim-3 mAb reversed this effect. These data clearly show that Tim-3 is actively involved in pregnancy maintenance through inhibition of TLR4-induced inflammatory responses.

We determined the transduction pathways involved in Tim-3-negatively regulated inflammatory reaction by TLR4 signal at the maternal-fetal interface. As the NF-κB pathway plays a critical role in LPS/TLR4-induced proinflammatory cytokine production^3^, we examined whether Tim-3 signaling suppressed LPS/TLR4-induced NF-κB activation. Because the ERK1/2 pathway was involved in Tim-3–mediated apoptosis inhibition, we analyzed whether interference with the ERK1/2 pathway also affected Tim-3/TLR4 crosstalk. To explore this, we first detected the activation of NF-κB in DSCs stimulated by LPS for different times, and found that LPS treatment up-regulated phosphor-NF-kB/total-NF-kB level in a time dependent manner (Figure S3A). Then DSCs were sequentially stimulated with Tim-3, LPS and U0126, and NF-κB phosphorylation was assessed again. As shown in Figure 4B, Tim-3 suppressed LPS-induced NF-κB phosphorylation; however, when U0126 was used to block the ERK1/2 pathway, Tim-3 no longer had this effect. In addition, we also examined whether LPS could activate ERK1/2 signal in DSCs. The results (Figure S3B) demonstrated that LPS stimulation did not increase ERK1/2 phosphorylation level. These data suggest that Tim-3 inhibits LPS-induced NF-κB activation via the ERK1/2 signal pathway.

### Tim-3 promoted Th2 bias at the maternal-fetal interface

**Figure 5:**
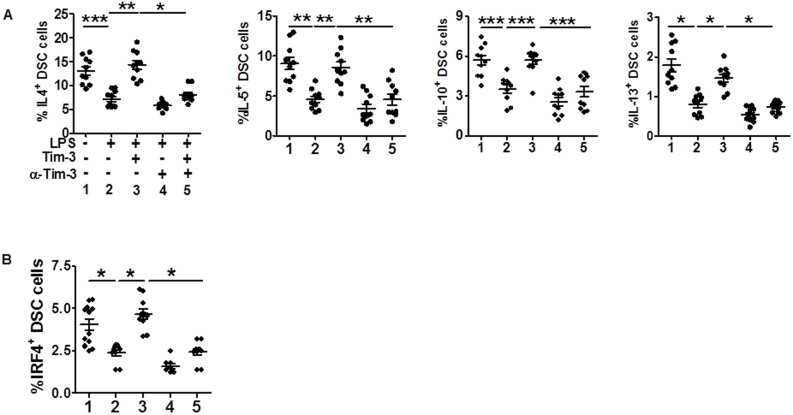
Tim-3 promotes Th2 bias at the maternal-fetal interface. (A and B) Flow cytometric analysis of production of the indicated cytokines (A) and IRF4^+^ (B) by DSCs after stimulation with or without LPS (10 ng/ml) in the presence or absence of Tim-3 or anti-Tim-3 mAb. Data represent mean ± SEM. n = 10 DSCs in the first trimester of normal pregnancy. *P < 0.5, **P < 0.01, ***P < 0.001.

It has been reported that TLR-induced pregnancy loss can be prevented by maternal administration of the immuno-regulatory cytokine, IL-10^3^,^21^. Given that Tim-3 suppressed LPS-induced pro-inflammatory cytokine production and that Tim-3^+^ DSCs produced higher levels of Th2 cytokines than Tim-3^−^ cells, we wondered whether Tim-3 affected Th2 cytokine production by DSCs upon LPS stimulation. Consistent with higher Th2 cytokine expression in Tim-3^+^ DSCs, treatment with Tim-3 released DSCs from LPS-induced inhibition of Th2 cytokine production. Blocking the Tim-3 signal with anti–Tim-3 mAb significantly down-regulated Th2 cytokine production by DSCs (Figure 5A). These data suggest that LPS stimulation results in declined Th2 cytokine production in addition to triggering apoptosis and pro-inflammatory reaction in DSCs. This may be another explanation for LPS induction of miscarriage.

IRF4 has been associated with Th2 bias^20^, and we have also shown increased IRF4 in Tim-3^+^ DSCs. Furthermore, DSCs treated with Tim-3 markedly up-regulated IRF4 expression, and DSCs treated with anti–Tim-3 mAb down-regulated IRF4 expression. These findings suggest that the Tim-3 signal induces basal Th2 bias by up-regulating the transcription factor IRF4 expression. We investigated whether IRF-4 is also involved in Tim-3/TLR4 crosstalk. The results in Figure 5B showed LPS reduced IRF4 expression in DSCs; Tim-3 significantly reversed this inhibitory effect, whereas anti–Tim-3 mAb dramatically inhibited IRF4 expression. Our data provide another possible mechanism by which Tim-3 promotes IRF4/Th2 cytokine production, thus preventing subsequent excessive immune activation.

**Figure 6:**
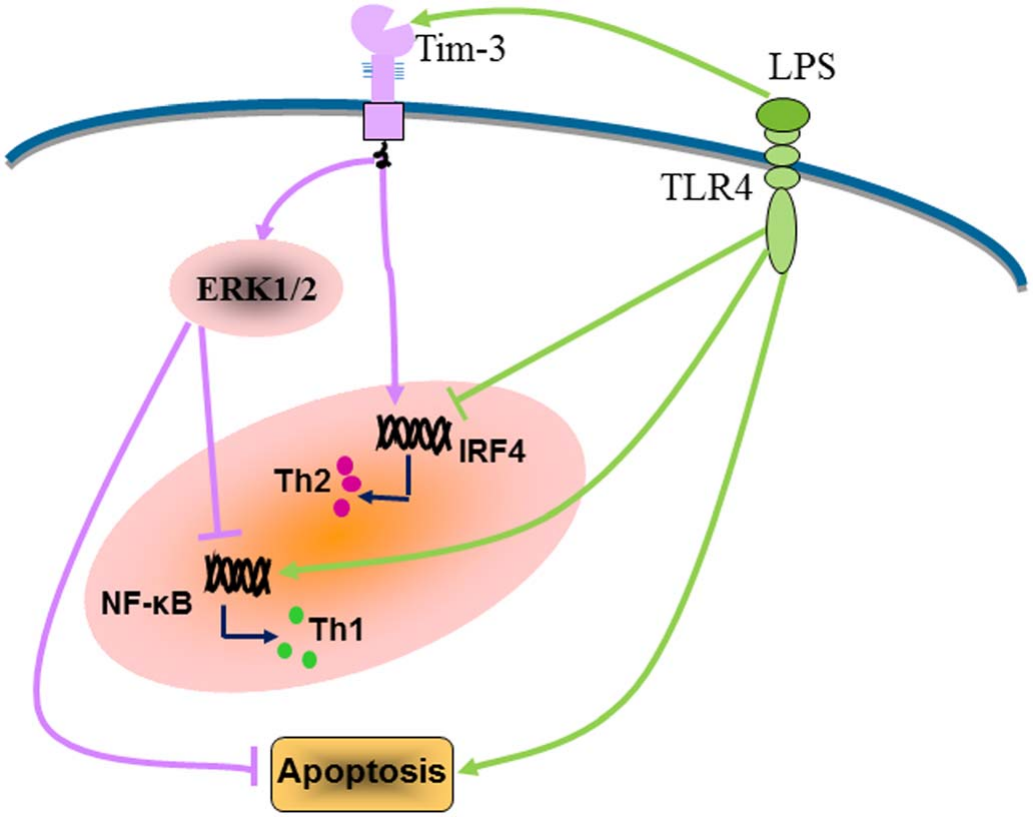
Schematic diagram of Tim-3–mediated negative regulation of the TLR response and promotion of Th2 bias at the maternal-fetal interface. (Left) LPS promotes Th1 bias at the maternal-fetal interface by activation of NF-κB and inhibition of IRF4. LPS can also induce apoptosis of DSCs. (Right) At the same time, LPS increases Tim-3 expression in DSCs, which in turn decreases DSC apoptosis and suppresses NF-κB activation mediated by LPS/TLR4 in an ERK1/2-dependent manner. Tim-3 not only increases basal IRF4 expression in DSCs but also abrogates LPS-induced IRF4 suppression, thus promoting Th2 bias at the maternal-fetal interface.

The proposal model for Tim-3–mediated negative regulation of the TLR response and induction of Th2 bias in the decidual stromal cells are illustrated in Figure 6.

## Discussion

In the present study, we provide the first evidence that Tim-3 is expressed by DSCs at the maternal-fetal interface. Interestingly, a notably higher level of Tim-3 was produced by DSCs from normal pregnancy than from miscarriage, which was associated with higher IRF4 expression and Th2 cytokine production by DSCs. Overshooting of activation of TLR signaling is associated with miscarriage. Although there was no difference in TLRs expression on DSCs between normal pregnancy and miscarriage, the enhanced Tim-3 expression might reduce TLR-trigged production of pro-inflammatory cytokines and DSC apoptosis through activation of the ERK1/2 pathway and inhibition of NF-κB activation. In addition, Tim-3 reversed the inhibitory effect of LPS on DSC Th2 cytokine production. Blockade of Tim-3 signaling by anti–Tim-3 mAb abolished the protective effects of Tim-3. Combined with our recent findings that blocking Tim-3 signaling with anti-Tim-3 antibody *in vivo* significantly increased embryo resorption while decreased litter size and inhibited fetal growth (unpublished data), this study indicated that Tim-3 might be important in the maintenance of normal pregnancy through forming a negative feedback loop to inhibit TLR-triggered inflammatory responses in DSCs (Figure 6). Approaches to promote Tim-3 signaling could be applied to protect the host from TLR-triggering of early pregnancy loss.

Decidua may be exposed to bacteria and viruses and provoke a substantial health threat to both mother and fetus. Thus, the decidua provides a first line of protection against invasion of external infectious agents. DSCs are the major cellular component of decidua. In addition to their traditional metabolic and supportive roles in pregnancy, DSCs also possess the capacity for tolerance of the fetal allograft and avoidance of excessive reaction to pathogens. From an immunological standpoint, the fetoplacental unit is an allograft tolerated by the maternal immune system. For years, the model of immune regulation during pregnancy has been based on the Th2 bias at the maternal-fetal interface. Increased expression of pro-inflammatory molecules in uterine has been reported in women suffering recurrent miscarriage^2^. The decidua is the primary site of production and secretion of immuno-regulatory factors^22^.

Tim-3 is one of the type I membrane proteins, which share a characteristic IgV, mucin, transmembrane, and cytoplasmic domain structure^14^. Tim-3 plays critical roles in both adaptive and innate immune regulation. The Tim-3 signal can induce Th1-type cell death and tolerance. Tim-3 is also constitutively expressed by macrophages and dendritic cells. Dysregulation of the Tim-3 signal has been linked to many diseases^23^,^24^,^25^,^26^. We were intrigued by the finding that Tim-3 expression in DSCs during normal pregnancy was 2-fold higher than that with miscarriage, indicating that the Tim-3 signal might play an essential role in pregnancy maintenance. Here, we found increased IRF4 expression in Tim-3^+^ DSCs, and that treatment of DSCs with Tim-3 markedly up-regulated IRF4 expression, whereas anti–Tim-3 mAb decreased IRF4 expression, which is important for Th2-type cytokine production^20^,^27^. Therefore, we directly examined Th2 cytokine production by Tim-3^+^ and Tim-3^−^ DSCs. Consistent with the pattern of IRF4 expression, Tim-3^+^ DSCs produced higher levels of IL-4, IL-10, IL-5, and IL-13. In addition, treatment with exogenous Tim-3 increased Th2 cytokine and reduced pro-inflammatory cytokine production, whereas treatment with anti–Tim-3 mAb reversed these effects (Figures 4A and 5A). Thus, the Tim-3 signal plays important roles in inducing a predominantly Th2-cytokine microenvironment at the maternal-fetal interface. Down-regulation of Tim-3 might be associated with miscarriage.

Mounting evidence suggests that spontaneous miscarriages are associated with a bias toward a pro-inflammatory cytokine profile^28^. TLRs are a family of innate pattern recognition receptors that can recognize pathogen-associated molecules and activate anti-pathogen responses^8^. Several studies have showed that TLR activation is associated with miscarriage^3^,^29^, and, therefore, we investigated the effects of TLRs on DSCs to further explore the correlation of TLR activation with pregnancy. We found that treatment of DSCs with different TLR ligands resulted in apoptosis and increments in IFN-γ and TNF-α production. LPS also promoted the expression of co-stimulatory molecule CD80 but not the inhibitory molecules, PDL-1 or PDL-2. Thus, TLR pathways might be involved in the formation of a pro-inflammatory microenvironment at the maternal-fetal interface.

Expression of Tim-3 and TLRs might be cross-regulated^16^,^18^. At the maternal-fetal interface, we found that TLR treatment resulted in incremental Tim-3 expression in DSCs. Tim-3 pretreatment diminished TLR-triggered apoptosis, whereas Tim-3 blockade exacerbated this effect. When Tim-3 and anti–Tim-3 mAb were administered at the same time, their effects on TLR-mediated apoptosis were reciprocally neutralized. Because spontaneous miscarriages are associated with a bias toward a pro-inflammatory cytokine milieu in which an uncontrolled TLR response plays a major pathogenic role, it is important to know whether the Tim-3 pathway is also involved in the regulation of LPS-triggered pro-inflammatory cytokine production. We found that Tim-3 actively suppressed the LPS-induced immune response, leading to down-regulation of IFN-γ, TNF-α and IL-1β production, and Tim-3 blockade significantly abrogated this down-regulation.

As Tim-3 does not contain obvious inhibitory signaling motifs^30^, little is known about Tim-3 signaling in immune cells, not to mention, in DSCs. Here, we showed that Tim-3 activated the ERK1/2 pathway, and ERK1/2 inhibitor U0126 markedly reversed Tim-3–induced apoptosis inhibition. ERK1/2 phosphorylation could activate multiple proliferation and survival-associated transcription factors. It could also repress the expression or activity of pro-apoptotic Bcl-2 family, ultimately resulting in cell apoptosis inhibition. Suppression of ERK1/2 activation could impair ERK1/2-mediated cell survival and induce cell apoptosis^31^,^32^. Therefore, we presume that Tim-3 protection of DSCs from apoptosis might depend on ERK1/2 signaling pathway. In addition, we also determined the intracellular cascade by which Tim-3 signaling inhibited the TLR response. We demonstrated that Tim-3 negatively regulates LPS/TLR4-induced pro-inflammatory response by inhibition of NF-κB activation. This pathway has also been reported in sepsis^16^. Although we observed that blockage of the ERK1/2 pathway eliminated NF-κB inhibition induced by Tim-3, the precise mechanism by which Tim-3-induced ERK1/2 activation inhibits TLR4 signaling remains obscure. A report has shown that ERK pathway negatively regulates NF-κB-driven transcription, in part, by inhibiting p38 MAP kinase activity^33^. We speculate that, in such a scenario, Tim-3 signaling, by a still unknown mechanism, enhance activation of ERK1/2, which would inhibit LPS-TLR4-induced NF-κB activation, then reduce NF-κB-driven production of pro-inflammatory cytokines by inhibiting p38 activity or other signal pathways in DSCs.

During pregnancy, a Th2 bias with an increased level of IL-4 and a decreased level of IFN-γ, may be generated at the maternal-fetal interface to protect the fetus from immunological attack by maternal immunocytes^34^. We have shown that Tim-3 protects DSCs from TLR-mediated apoptosis and pro-inflammatory responses. We found that, apart from triggering pro-inflammatory cytokine production, LPS also reduced the generation of Th2 cytokines. Treatment of DSCs with Tim-3 significantly enhanced IL-4, IL-10, IL-5, and IL-13 production, which were decreased after LPS stimulation and following Tim-3 blockade. In addition, LPS down-regulated IRF4 expression, whereas Tim-3 reversed the inhibitory effect of LPS on IRF4 expression in DSCs. However, Tim-3 blockade strikingly inhibited basal IRF4 expression, and anti–Tim-3 antibody treatment exacerbated LPS-mediated suppression of IRF4. Tim-3 usually serves as a receptor, so exposure of cells to exogenous Tim-3 may inhibit Tim-3 signal transmission as exogenous soluble Tim-3 ligated its nature ligands (e.g., galectin-9). It has been reported that external soluble Tim-3 blocked the inhibitory effect of Tim-3^35^,^36^. Based on this, treatment of DSC cells with Tim-3 should augment the pro-inflammatory responses induced by the TLR signal; however, we observed contradictory results. This may due to the direct interaction of exogenous Tim-3 and the putative Tim-3 ligand galectin-9, which was highly expressed in DSCs (data not shown). A study by Leitner et al. reported that Tim-3 did not act as a receptor for galectin-9^37^, but HMGB1, another ligand of Tim-3^38^ was also expressed in abundant cytoplasmic of decidual cells^39^. Still it has not been shown whether soluble Tim-3 Ig mimics the function of native soluble Tim-3. Therefore, we came to the conclusion that, at the maternal-fetal interface, treatment with exogenous soluble Tim-3 did not facilitate, but suppressed, TLR-mediated apoptosis and pro-inflammatory reaction of DSCs. Nonetheless, further experiments are still needed to identify the mediators responsible for the observed results.

In conclusion, our data provide new evidence demonstrating the key role of Tim-3 in the maternal-fetal tolerance via non-classical immune cells, and highlight a role for Tim-3 in a negative feedback loop to inhibit TLR-mediated apoptosis and pro-inflammatory reaction of DSCs. These findings identify Tim-3 as a key regulator of DSC function, which might be critical for normal pregnancy.

## Methods

### Samples

First-trimester (gestational age 6–12 weeks) human decidual tissues were obtained from clinically normal pregnancies (terminated for non-medical reasons, n = 98) and miscarriages (diagnosed as recurrent spontaneous abortion and excluding endocrine, anatomic, and genetic abnormalities; infection; and poor health habits; n = 43). We checked the pregnancy with ultrasound, test of hCG-β and progesterone levels from peripheral blood. The miscarried decidual tissues were obtained very soon after they are diagnosed. The cultured decidual cells from normal pregnancies and miscarriages were confirmed to be vial through trypan blue staining before each experiment. The Human Research Ethics Committee of the Obstetrics and Gynecology Hospital of Fudan University approved the collection and use of the samples. All participants provided written informed consent. All the methods were carried out in accordance with the approved guidelines.

### Isolation and culture of DSCs

DSCs were isolated by collagenase IV/DNase-I digestion and discontinuous Percoll gradient centrifugation as our previously described^40^,^41^. This method supplies a 99% purity of DSCs (Vimentin^+^ CK7^−^ Smooth actin^−^ Factor VIII^−^). Vimentin was used as a marker of DSCs in the flow cytometric analysis.

### Treatment of DSCs

Freshly isolated DSCs were cultured overnight in complete medium and further incubated in serum-free medium for 12 h, followed by stimulation with a range of concentrations of the TLR ligands peptidoglycan from *Staphylococcus aureus* (pGN-SA; 08K03-SV, InvivoGen, San Diego, CA, USA), poly(I:C) (11C21-MM, InvivoGen), and LPS (13I06-MM, InvivoGen) for 48 h. DSCs were collected for flow cytometric analysis. To some wells were added 1000 ng/ml recombinant Tim-3 (2365-TM-050, R&D Systems, Minneapolis, MN, USA), 10 μg/ml anti–Tim-3 mAb (clone F38-2E2, Biolegend, San Diego, CA, USA), JNK signaling pathway inhibitor SP600125 (30 μM), P38 signaling pathway inhibitor SB202190 (30 μM), NF-κB inhibitor BAY 11-7082 (30 μM), or MEK1/2 inhibitor U0126 (30 μM).

### Quantification of Tim-3 mRNA by PCR

Total RNA was extracted from DSCs and reverse transcribed. Complementary DNA was amplified by real-time PCR in a final volume of 50 μl containing 25 μl of Hot-Start PCR Master Mix (RuiCheng Bio) and 200 nM of each primer probe. The primers in the study were as follows: human Tim-3 (127 bp), forward primer: 5′-ACC AGC CAA GGT CAC CCC T-3′, reverse primer: 5′-ATT TAT ATC AGG GAG GCT CCC C-3′ and human GAPDH (138 bp), forward primer: 5′-GCA CCG TCA AGG CTG AGA AC-3′, reverse primer: 5′-TGG TGA AGA CGC CAG TGG A-3′ (Shenggong Corp., Shanghai, China). Each sample was analyzed in duplicate using an ABI Prism™ 7000 Sequence Detector (Applied Biosystems, Carlsbad, CA, USA).

### Immunohistochemical analysis

Sections (5-mm) of paraffin-embedded first trimester human decidua were rehydrated in Tris-buffered saline (TBS), and incubated with hydrogen peroxide and 1% bovine serum albumin (BSA) in TBS to block endogenous peroxidase activity. Sections were incubated with goat anti-human Tim-3 antibody (0.5 mg/ml, ab47997; Abcam, Cambridge, UK) overnight in a humidified chamber. After washes, sections were overlaid with poly-HRP anti-goat IgG (Golden Bridge International, Beijing, China). The reaction was developed with 3, 3-diaminobenzidine (DAB) and sections were counterstained with hematoxylin.

### Flow cytometry

Expression of cell surface molecules and intracellular cytokine were evaluated using flow cytometry. A minimum of 10,000 events was acquired using a Beckman-Coulter (Brea, CA, USA) CyAn ADP flow cytometer and analyzed with FlowJo software (Tree Star, Ashland, OR, USA). Fluorescein isothiocyanate (FITC)-conjugated anti-human Vimentin, phycoerythrin (PE)-conjugated anti-human Tim-3(Biolegend), PE-conjugated anti-human PDL-1 or active Caspase-3, allophycocyanin (APC)-conjugated anti-human PDL-2, (BD, San Jose, CA, USA), FITC-conjugated anti-IFN-γ or IL-1β, PE-conjugated anti–Vimentin, PE/CY7-conjugated anti-CD80 or IL-10 or TNF-α, APC-conjugated anti-CD86 or IL-5 or IL-13, Brilliant Violet 421-conjugated anti–IL-2 or –IL-4 (Biolegend) and anti-human/mouse IRF4 eFluor® 660 (eBioscience, San Diego, CA, USA). For intracellular staining, cells were fixed and permeabilized using the Fix/Perm Kit (Biolegend). Cell fluorescence was measured using a Beckman-Coulter CyAN ADP flow cytometer and analyzed with FlowJo software (Tree Star). Murine IgGs of the same isotype were used as negative controls.

### Annexin V and propidium iodide (PI) staining for cell apoptosis

Freshly isolated DSCs were seeded at 2 × 10^5^ cells/well in 24-well plates; incubated overnight; and treated with TLR ligands, Tim-3, anti-Tim-3, and signal transduction inhibitors described above. The cells were harvested and resuspended in 100 μl annexin-binding buffer containing 5 μl FITC-annexin V and 1 μl PI working solution (BD Biosciences, Franklin Lakes, NJ, USA) and then incubated in the dark for 15 min at room temperature. An additional 400 μl binding buffer was added, and DSCs were analyzed immediately by flow cytometry (BD Biosciences).

### In-cell western assay

In-cell western analysis was carried out to determine intracellular levels of NF-κB, ERK1/2, and the phosphorylated forms of NF-κB and ERK1/2, according to the protocol described by Egorina^42^ and our previous procedure^43^. Primary cultures of DSCs in 96-well plates were cultured in serum-free medium for 12 h and then stimulated with 1 μg/ml Tim-3 for 0, 30, 60, 90, or 120 min, or 10 ng/ml LPS for 0, 30, 60, 90, 120, 150 or 180 min followed by fixation, infiltration, and blocking. The cells were incubated with rabbit anti-human ERK1/2 (1:50) and mouse anti-human phospho-ERK1/2 (1:50) antibodies at 4°C overnight. The second IRDye700DX®-conjugated affinity-purified (red fluorescence) anti-mouse antibody and IRDye800DX®-conjugated affinity purified (green fluorescence) anti-rabbit antibody (both from Rockland, Gilbertsville, PA, USA) were used. Images of target molecule fluorescence were obtained using the Odyssey Infrared Imaging System (LI-COR Biosciences). Expression of phosphorylated proteins was calculated as the ratio of the intensity of staining of phosphorylated protein to that of the corresponding total protein.

To examine NF-κB activation level, DSCs cultured in 96-well plates were sequentially stimulated with Tim-3, LPS, and U0126. NF-κB phosphorylation was assessed using mouse anti-human NF-κB p65 (1:50) and rabbit anti-human phospho–NF-κB p65 (Ser 311) (1:50). These experiments were carried out in triplicate and repeated three times.

### ELISA

DSCs (2 × 10^5^/well) were grown in 24-well plates in the presence of 10 ng/mL LPS for 24 h, 48 h and 72 h. There-after, the levels of secreted TNF- α and IFN-γ in the supernatant from each experiment were quantified by using the commercially available ELISA kit (R&D System) following the manufacturer's instructions.

### DAPI staining

Freshly isolated DSCs were grown on BD Falcon™ culture slides and exposed to different concentrations of LPS for 48 h. At the end of treatment, the culture media were removed, and the cells were permeabilized with 0.1% Triton X-100 after being fixed with 4% paraformaldehyde. Thereafter, the cells were stained with 4-6-diamidino-2-phenylindole (DAPI) (1:200; Invitrogen). Fluorescence images were observed by using an Olympus BX51 fluorescence microscope (Tokyo, Japan), and recorded with a high-resolution DP70 Olympus digital camera. The percentage of apoptosis was determined by nuclear morphology. At least 400 cells were counted in each group with the counter ‘blinded' to sample identity to avoid experimental bias.

### Statistical analysis

Significance of differences between two groups was determined by post hoc Dunnett t test. Multiple groups were analyzed by one-way or two-way ANOVA with Bonferroni post tests using Prism Version 5 software (GraphPad, San Diego, CA, USA). For all statistical tests, p values < 0.05 were considered statistically significant.

## Author Contributions

S.C.W. carried out experiments. S.C.W. and M.R.D. conceived experiments and analyzed data. C.M.C., X.M.Z., Y.H.L., H.L.P., Y.T., D.Z., C.S., R.Z., Y.W. and M.M.Y. coordinated the sample collection, data interpretation, literature search, and figure preparation. S.C.W. drafted the manuscript. C.M.C., X.M.Z., D.J.L. and M.R.D. revised the manuscript. All authors reviewed the manuscript.

## Supplementary Material

Supplementary Information Supplementary information
