# Supplementary material for: Tim-3 protects decidual stromal cells from toll-like receptor-mediated apoptosis and inflammatory reactions and promotes Th2 bias at the maternal-fetal interface
Source: Sci Rep. 2015 Mar 11;5:9013. doi: 10.1038/srep09013 (PMC4355741; doi:10.1038/srep09013)
Supplement: Supplementary Information [file srep09013-s1.pdf]

Tim-3 protects decidual stromal cells from toll-like receptor-mediated apoptosis and inflammatory reactions and promotes Th2 bias at the maternal-fetal interface

Running title: The roles of Tim-3 signal in decidual stromal cell

SongCun Wang1, †, ChunMei Cao, 2, †, HaiLan Piao1, YanHong Li1, Yu Tao1, XiaoMing Zhang3, Di Zhang1, Chan Sun1, Rui Zhu1, Yan Wang1, MinMin Yuan1, DaJin Li1, Mei-Rong Du1,*

Figure S1. Poly(I:C) and pGN-SA induced apoptosis and proinflammatory cytokine production in DSCs.

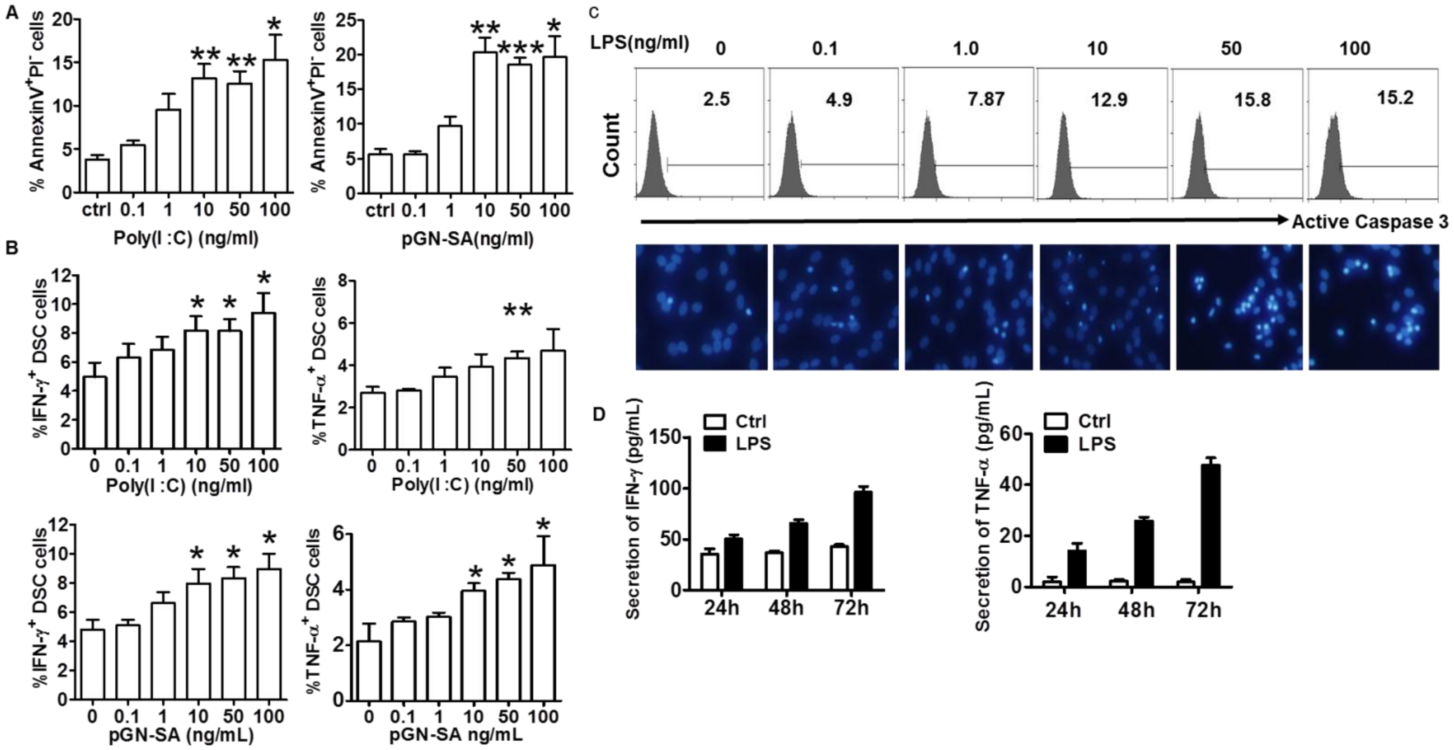

Quantitation of flow cytometric analysis of apoptosis based on annexin expression and PI staining (A) and IFN-γ and TNF-α production (B) in DSCs treated for 48 h with the indicated concentrations of poly(I:C) and pGN-SA. (C) Flow cytometric analysis of active caspase-3 (upper) and DAPI staining of DSC nuclear morphology after treatment of DSCs with the indicated concentrations of LPS. Flow cytometry plot shown is from one representative experiment; (D) The secretion of IFN-γ (left) and TNF-α (right) by DSCs in the treatment of LPS or control for different times. Data represent mean ± SEM of triplicates of one representative experiment of five separate experiments. Data represent mean ± SEM. n=14 DSCs in the first trimester of normal pregnancy. *P≤0.05, **P≤0.01, ***P≤0.001.

Figure S2. Tim-3 protected DSCs from Poly (I:C)- and pGN-SA–mediated apoptosis

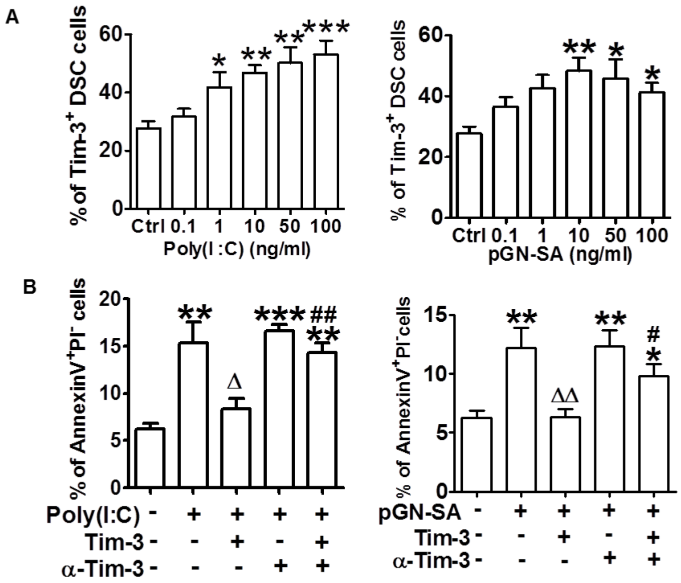

Quantitation of flow cytometric analysis of (A) Tim-3 expression in DSCs after treatment with the indicated concentrations of poly (I:C) and pGN-SA for 48 h, and of (B) apoptosis based on annexin expression and PI staining in DSCs treated with or without 10 mg/ml poly(I:C) (left) or pGN-SA (right) in the presence or absence of Tim-3 or anti－Tim-3 mAb. Data represent mean ± SEM. *P≤0.05, **P≤0.01, ***P≤0.001.

Figure S3. LPS activates NF-κB but not ERK1/2 in DSCs

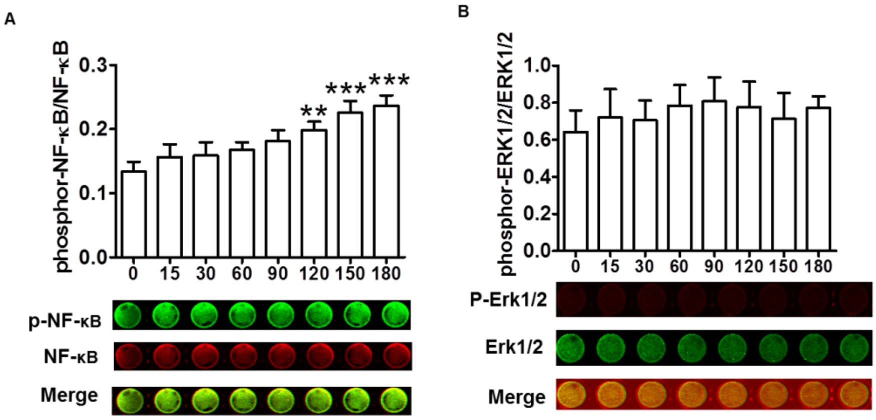

In-cell western analysis (lower) and quantitation (upper) of phosphorylation/activation of (A) NF-κB and (B) ERK1/2 in DSCs stimulated with or without LPS for different times. Images are representative of eight individual experiments. **P<0.01, ***P<0.001, compared with group 0.
